# Differential Predictive Roles of A- and B-Type Nuclear Lamins in Prostate Cancer Progression

**DOI:** 10.1371/journal.pone.0140671

**Published:** 2015-10-15

**Authors:** Irena Saarinen, Tuomas Mirtti, Heikki Seikkula, Peter J. Boström, Pekka Taimen

**Affiliations:** 1 Department of Pathology, University of Turku and Turku University Hospital, Turku, Finland; MediCity, Research Laboratory, University of Turku, Turku, Finland; 2 Department of Pathology, Helsinki University Hospital and Finnish Institute for Molecular Medicine, University of Helsinki, Helsinki, Finland; 3 Department of Urology, Turku University Hospital, Turku, Finland; RWTH Aachen, GERMANY

## Abstract

**Background:**

Prostate cancer (PCa) is the most common cancer among men in western countries. While active surveillance is increasingly utilized, the majority of patients are currently treated with radical prostatectomy. In order to avoid over-treatment, there is an indisputable need for reliable biomarkers to identify the potentially aggressive and lethal cases. Nuclear intermediate filament proteins called lamins play a role in chromatin organization, gene expression and cell stiffness. The expression of lamin A is associated with poor outcome in colorectal cancer but to date the prognostic value of the lamins has not been tested in other solid tumors.

**Methods:**

We studied the expression of different lamins with immunohistochemistry in a tissue microarray material of 501 PCa patients undergoing radical prostatectomy and lymph node dissection. Patients were divided into two staining categories (low and high expression). The correlation of lamin expression with clinicopathological variables was tested and the association of lamin status with biochemical recurrence (BCR) and disease specific survival (DSS) was further analyzed.

**Results:**

Low expression of lamin A associated with lymph node positivity (p<0.01) but not with other clinicopathological variables and low expression had a borderline independent significant association with DSS (HR = 0.4; 95% CI 0.2–1.0; p = 0.052). Similarly, low lamin C expression associated with poorer survival (HR = 0.2; 95% CI 0.1–0.6; p = 0.004). Lamin B1 expression did not associate with clinicopathological variables but high expression independently predicted BCR in multivariable Cox regression analysis (HR = 1.8; 95% CI 1.1–2.9; p = 0.023). Low expression of lamin B2 correlated with lymph node positivity (p<0.01) and predicted unfavorable DSS (HR = 0.4; 95% CI 0.2–1.0; p = 0.047).

**Conclusions:**

These results suggest differential roles for lamins in PCa progression. Reduced amounts of lamin A/C and B2 increase risk for lymph node metastasis and disease specific death possibly through increased nuclear deformability while high expression of lamin B1 predicts disease recurrence.

## Introduction

Prostate cancer (PCa) is the most common malignancy among men and a remarkable public health challenge in Western countries. In United States, more than 230 000 new cases and 29 000 PCa related deaths was expected to be diagnosed in 2014 [1]. The major risk factors for PCa include age, positive family history and African-American race [2]. The majority of patients are currently treated with radical prostatectomy and/or radiotherapy while active surveillance may be beneficial for low risk patients. In most of the cases, the disease is local and only ~7% of patients treated with radical prostatectomy (RP) die from PCa during a 15-year follow-up [3]. Furthermore, it seems clear that the treatment of low-grade cancer (Gleason score 6 or below) confers no survival benefit when compared to other causes of death. The use of prostate-specific antigen (PSA) has revolutionized the diagnostics of PCa during the past 25 years and more recently systematic PSA screening has been shown to decrease PCa-related mortality [4]. However, PSA lacks the specificity to detect clinically significant cancers and the drawback is over-diagnosis of cancers that would not have effect on the quality and expectancy of life if left untreated [5]. This in turn leads to overtreatment with potential side effects. In order to avoid over-treatment, there is an indisputable need for reliable biomarkers to identify the potentially aggressive and lethal cases from those that remain local.

Nuclear lamins are type V intermediate filament (IF) proteins. They are the major components of the nuclear lamina, a fibrous meshwork of proteins that underlies the inner nuclear membrane [6]. Lamins are devided into two subgroups, A-type and B-type lamins. A single gene, *LMNA*, encodes all A-type lamins that include lamin A, lamin C, a testis-specific isoform lamin C2 and minor isoform lamin AΔ10. The major B-type lamins, lamin B1 and lamin B2, are encoded by two genes (*LMNB1* and *LMNB2*, respectively) [7]. In addition, a germ cell specific lamin B3 is generated from *LMNB2* through alternative splicing [8]. At least one B-type lamin is expressed in all metazoan cells, while A-type lamins are primarily expressed in differentiated cells [7].

The lamins have multiple functions in the cell nucleus. They provide mechanical support for the nucleus and play a role in DNA replication and repair, transcription, chromatin organization and gene regulation [7]. Numerous mutations in *LMNA* cause a group of inherited human diseases or disorders commonly called the laminopathies. These include, for example, cardiac and skeletal myopathies, lipodystrophies, peripheral neuropathy and premature aging disease known as progeria syndrome [9]. While the fundamental pathobiology of these diseases is mostly poorly understood, abnormalities of nuclear shape (e.g. herniations and lobulations) are commonly reported in cells carrying disease-associated lamin mutations. Interestingly, similar morphological changes of the nucleus are frequently observed in cancer cells including PCa cells [10]. Therefore, it is possible that altered expression and modifications in lamins contribute malignant transformation in cancer cells [11].

Only a few retrospective studies have substantiated the association between altered lamin expression and patient outcome. In stage I-III colorectal cancer (CRC), the patients with lamin A expressing tumors had significantly increased risk to die from CRC when compared to patients with lamin A negative tumors [12]. Based on *in vitro* studies, the authors further concluded that this is due to increased cell motility, invasiveness and stem cell-like phenotype promoted by lamin A/C. On the contrary, Belt et al. reported that loss of lamin A/C in stage II-III colon cancer is associated with disease recurrence [13]. In nodal diffuse large B-cell lymphoma inactivation of lamin A/C gene by CpG island promoter hypermethylation is associated with poor survival [14].

The role of lamins in PCa has been studied more recently. Using mass spectrometry (MS) and immunohistochemistry, Skvortsov et al. reported decreased expression of lamin A in low grade (Gleason score 6) tumors relative to paired samples from histologically normal tissue while an increased expression was detected in high risk (Gleason score 8) tumors [15]. Furthermore, lamin A/C is overexpressed at the invasive front of PCa tissue and promotes cell growth, migration and invasion through the PI3K/AKT/PTEN pathway under culture conditions [16]. The expression of B-type lamins, on the other hand, is increased in cancerous prostate tissue and strongly correlates with Gleason score [17]. MS analysis further suggests that lamin B1 is hyperphosphorylated in androgen independent PC-3 cells [18]. Interestingly, chromosomal regions associated with PCa risk localize to nuclear lamin B-deficient microdomains (LDMD) that exhibit reduced gene transcription (10). While the nature of these structures is currently unclear, the frequency of LDMDs correlates with PCa cell line aggressiveness, cell motility and Gleason grade [10]. In summary, lamins undergo significant alterations during malignant transformation but to date it is unclear whether any of these changes have predictive value in PCa, especially in high grade cancer.

In the present study we studied the expression of different lamins in PCa in detail using tissue microarray (TMA) material covering over 500 patients treated with radical prostatectomy and lymph node dissection. Our results show that decreased expression of A-type lamins is associated with elevated risk for lymph node metastasis and disease specific death while increased expression of lamin B1 is associated with BCR and local spread. Additionally, low lamin B2 expression predicted higher risk of PCa mortality. We also discuss the potential cell biological mechanisms and their significance for clinical risk stratification.

## Materials and Methods

### Tissue material

Paraffin embedded formalin fixed tissue material from 501 patients operated with radical prostatectomy and limited pelvic lymphadenectomy in the Turku University Central Hospital between January 2000 and September 2005 were used for constructing the TMA blocks as previously described [19]. In brief, three to twelve (median 3.0) adjacent cores of 1 mm in diameter were obtained from the index carcinoma lesion that was considered the most significant (based on the Gleason grade pattern, the volume of cancer lesion and/or extra-prostatic extension). In addition, one core from histologically benign prostate tissue was obtained from the same patient. These tissue cores were transferred from the donor blocks to the recipient TMA blocks that contained in total 63 cores per block including control tissues from normal liver. All cases in the final TMA blocks were graded based on the International Society of Urological Pathology updated Gleason grading system [20]. At the time of surgery, no consent was obtained from the participants for this specific study. At the time of the study, the use of tissue material and the corresponding clinicopathological and follow-up data was approved by the Ethics Committee of the Hospital District of the Southwest Finland (130/180/2008) and the National Authority for Welfare and Health (Valvira 394/05.01.00.06/2009) according to the national legislation. Patient information was anonymized and de-identified prior to analysis.

### Clinical data

None of the patients received androgen deprivation or radiotherapy prior to or immediately after the surgery. The follow-up was conducted by digital rectal examination and post-operative PSA measurement at least three times during the first year after the surgery and at least once a year during the following years. Of the 501 patients, 158 (31%) had a biochemical recurrence (BCR) defined as PSA level of 0.2 μg/L or above after the surgery.

The staging of the PCas was performed according to the WHO pTNM classification system [21]. 28 patients (4.4%) died from PCa during the follow-up, while 39 (7.8%) died of other causes. Due to long time interval between operations, each patient’s original clinical Gleason score (GS) was re-evaluated by experienced genitourinary pathologist according to the current Gleason grading system criteria [20] using hematoxylin-eosin sections from the original radical prostatectomy specimens. This score was used in the survival analyses and is presented in Table 1.

**Table 1:** Clinicopathological variables of the patient cohort (n = 501).

Variable		Number of patients
Age		
	<60 years	169 (34%)
	60–65 years	187 (37%)
	>65 years	143 (29%)
PSA group (μg/L)		
	<10	306 (61%)
	10–20	116 (23%)
	>20	38 (8%)
pT-category^a^		
	pT2	209 (42%)
	pT3a	160 (32%)
	pT3b	52 (10%)
Gleason group		
	<7	181 (36%)
	7	199 (40%)
	>7	96 (19%)
Extraprostatic extension		
	Yes	203 (41%)
Surgical margin		
	Positive	190 (38%)
Lymph node status		
	N+	21 (4%)
BCR		
	Yes	157 (31%)
	Alive	444 (89%)
	Death due Pca	20 (4%)
	Death, other	37 (7%)
Follow-up time (months)		
	Median	91
	Range	1–167

^a^ Pathological T (pT) category refers to the stage of primary tumor in the TNM tumor staging system. pT2 = Tumor confined within prostate, pT3a = Extracapsular extension, pT3b = tumor invading seminal vesicle(s).

### Immunohistochemistry and microscopy

In order to perform immunohistochemical (IHC) assessment, paraffin was first removed with xylene and the sections were rehydrated with graded series of alcohol. All the antibodies were tested on prostate TMA test blocks prior to use in the final TMA material in order to optimize the dilution and the most suitable pre-treatment. Epitope unmasking was carried out by microwaving the tissue slides in either Tris-HCl buffer (pH 9; lamin B1, B2 and C antibodies) or Citrate based buffer (pH 6; lamin A antibody) for 10 min. The primary antibodies used were mouse monoclonal anti-lamin A (1:1000, clone 133A2, Abcam), goat polyclonal anti-lamin B1 (1:4000, C-20, Santa Cruz), mouse monoclonal anti-lamin B2 (1:500, LN43, Abcam) and rabbit polyclonal anti-lamin C (1:150, RalC, Novus). Due to variation in the staining between different patches of lamin C antibody, only 199 patients stained with the same patch were included in the final statistical analysis. The stainings were carried out using Ventana automated staining machine and the primary antibodies were detected with Vectastain anti-mouse or anti-goat HRP-conjugated secondary antibodies. The slides were counterstained with hematoxylin and observed with Olympus BX60 microscope (Olympus Optical Co., Ltd., Tokyo, Japan).

The TMA slides were graded visually for IHC grades, without the knowledge of clinicopathological characteristics of the patients. The staining intensities of each core were graded as 0 (no specific staining in carcinoma cells, intense staining in benign glands and stromal cells), 1 (low intensity nuclear lamina staining in carcinoma cells, more intense staining in benign glands or stromal cells), 2 (moderate intensity and clearly visible lamina in carcinoma cells, yet more intense staining in benign glands and stromal cells) or 3 (high intensity lamina staining equivalent to, or exceeding the intensity of normal glands and stromal cells). Patients whose tissue samples were stained poorly (based on internal controls) or detached from the slides during staining process were excluded from the analysis. The mean intensity values of 3 or more carcinoma cores were determined for each patient and eventually the patients were dichotomized into low (IHC grade 0 and 1) and high (IHC grades 2 and 3) expressing groups for further statistical analysis (except for lamin B1 and C, see Table 2). Dichotomization was based on the distribution of different subgroups for each lamin staining in order to have comparable groups with sufficient number of patients (see Table 2).

**Table 2:** The distribution of expression for each lamin in the patient cohort.

		Number of patients		
Marker	GS* group	Low expression	High expression	Total
Lamin A		IHC grade 0–1	IHC grade 2–3	
	Entire group	251 (52.0%)	232 (48.0%)	483
	Subgroup (GS>6)	141 (50.0%)	141 (50.0%)	282
Lamin C		IHC grade 0	IHC grade 1–3	
	Entire group	41 (22.9%)	138 (77.1%)	179
	Subgroup (GS>6)	22 (21.4%)	81 (78.6%)	103
Lamin B1		IHC grade 0–2	IHC grade 3	
	Entire group	142 (30.3%)	327 (69.7%)	469
	Subgroup (GS>6)	82 (30.0%)	191 (70.0%)	273
Lamin B2		IHC grade 0–1	IHC grade 2–3	
	Entire group	169 (37.5%)	282 (62.5%)	451
	Subgroup (GS>6)	88 (33.5%)	175 (66.5%)	263

*GS = Gleason score

### Statistical analysis

The statistical analysis was performed with SPSS 20 (IBM). The correlations between clinicopathological variables and biomarkers were analyzed using chi-square test. The correlation between mean lamin expression in benign and cancerous prostate tissue from the same patients were analyzed with independent-samples T test. The Kaplan-Meier method, Log-rank test and Cox proportional hazards regression model were used to analyze the association of staining intensity to outcome. For outcome analyses, the time for BCR-free survival was calculated from the day of surgery to the day of detection of PSA of 0.2 μg/L or above. The disease specific survival time was calculated from the date of surgery to the date of the last follow-up visit or death.

The survival analyses were performed for the entire cohort (except for lamin C, see above) and for a high risk subpopulation including patient with GS 7–10 tumors. All statistical tests were two-sided and p-values ≤0.05 were considered statistically significant.

## Results

In order to study whether expression of different lamins is associated with PCa biochemical progression and disease specific survival (DSS), TMA material containing at least triplicate cancerous samples and one morphologically benign sample from 501 radically operated PCa patients was stained with antibodies detecting different lamins (A, C, B1 and B2) and the staining intensities were compared to clinicopathological variables. The demographics of patients are presented in the Table 1. In the Kaplan-Meier estimation analysis, the well-known clinicopathological features such as high GS and preoperative PSA, T category, extraprostatic extension (EPE) and seminal vesicle invasion (SVI) were statistically significantly associated with BCR indicating that our cohort is highly representative to test the correlation between the proteins of interest and patient outcome (data not shown). Similarly, high GS, T category, positive surgical margin, EPE, SVI and lymph node (LN) positivity predicted shortened time for disease-specific death (data not shown). In multivariable Cox regression analysis that took other prognostic markers into account, high GS, preoperative PSA and T-category were independent predictors of BCR (Table 3) while high GS, T-category and LN positivity predicted death from PCa (Table 4).

**Table 3:** Uni- and multivariate Cox regression analysis for risk of biochemical recurrence. In addition to entire study cohort, a subpopulation of patients with GS>6 tumor was analyzed separately.

		Entire group						Subgroup (GS>6)					
		Univariate			Multivariate			Univariate			Multivariate		
Variable		HR	95% CI	p-value	HR	95% CI	p-value	HR	95% CI	p-value	HR	95% CI	p-value
Gleason score													
	<7		ref			ref			na			na	
	7	1.9	1.2–3.0	<0.003*	1.3	0.8–2.3	0.32		ref			ref	
	>7	4.8	3.0–7.5	<0.001*	2.6	1.5–4.7	0.001*	2.4	1.7–3.5	<0.001*	2.0	1.0–3.3	<0.002*
sPSA													
	Continuous	1.06	1.05–1.08	<0.001*	1.04	1.02–1.06	<0.001*	1.05	1.04–1.07	<0.001*	1.04	1.02–1.06	<0.001*
pT category^a^													
	pT2		ref			ref			ref			ref	
	pT3	3.1	2.1–4.6	<0.001*	2.2	1.3–3.5	0.002*	2.3	1.4–3.7	0.001*	1.7	1.0–2.9	0.053
Lamin A													
	Low		ref			ref			ref			ref	
	High	1.0	0.7–1.3	0.85	0.81	0.5–1.2	0.29	0.9	0.6–1.3	0.58	0.7	0.5–1.1	0.15
Lamin B1													
	Low		ref			ref			ref			ref	
	High	1.5	1.0–2.2	0.04*	1.8	1.1–2.9	0.023*	1.7	1.0–2.7	0.036*	1.6	0.9–2.9	0.077
Lamin B2													
	Low		ref			ref			ref			ref	
	High	1.1	0.8–1.6	0.48	0.95	0.6–1.4	0.81	0.95	0.6–1.4	0.82	0.85	0.5–1.4	0.49
Lamin C													
	Low		ref			ref			ref			ref	
	High	1.0	0.5–1.9	0.88	0.70	0.3–1.7	0.41	1.1	0.5–2.5	0.9	0.58	0.2–1.7	0.32

HR, hazard ratio (describes the relative risk of the event at any given time based on comparing the group with the reference group); 95% CI, 95% confidence interval (A 95% confidence interval means that 95% of the values of an unobservable parameter of interest would set in the interval if the experiment is repeated); na, not available; ref, reference

* Significant p-value

^a^ Pathological T category refers to the stage of primary tumor in the TNM tumor staging system. pT2 = Tumor confined within prostate, pT3 = Tumor invading extraprostatic tissue and/or seminal vesicle(s).

**Table 4:** Uni- and multivariate Cox regression analysis for risk of prostate cancer-specific death. In addition to entire study cohort, a subpopulation of patients with GS>6 tumor was analyzed separately.

		Entire group						Subgroup (GS>6)					
		Univariate			Multivariate			Univariate			Multivariate		
Variable		HR	95% CI	p-value	HR	95% CI	p-value	HR	95% CI	p-value	HR	95% CI	p-value
Gleason score													
	<7		ref			ref			na			na	
	7	8.8	1.0–71.0	0.042*	9.3	1.1–77.4	0.039*		ref			ref	
	>7	28.9	3.7–224.7	0.001*	37.6	4.8–296.2	0.001*	3.3	1.4–8.0	0.008*	3.0	0.9–9.3	0.063
sPSA													
	Continuous	1.80	0.99–3.24	0.051				1.02	0.98–1.07	0.31			
pT category^a^													
	T2		ref						ref				
	T3	60.3	1.4–2688.4	0.034*				41.4	0.6–2642.6	0.079			
N category^b^													
	N0								ref			ref	
	N1							6.5	2.5–16.9	<0.001*	5.5	2.1–14.6	0.001*
Lamin A													
	Low		ref			ref			ref			ref	
	High	0.3	0.2–1.5	0.27	0.4	0.16–1.0	0.052	0.4	0.1–1.0	0.052	0.4	0.2–1.2	0.11
Lamin B1													
	Low		ref			ref			ref			ref	
	High	1.5	0.4–5.1	0.55	1.6	0.5–5.7	0.44	1.4	0.4–5.1	0.57	1.7	0.5–6.1	0.40
Lamin B2													
	Low		ref			ref			ref			ref	
	High	0.5	0.2–1.2	0.11	0.41	0.17–0.99	0.047*	0.4	0.2–1.0	0.058	0.6	0.2–1.6	0.29
Lamin C													
	Low		ref			ref			ref			ref	
	High	0.4	0.2–1.2	0.095	0.2	0.1–0.6	0.004*	0.3	0.1–0.8	0.023*	0.3	0.1–0.9	0.03*

HR, hazard ratio (describes the relative risk of the event at any given time based on comparing the group with the reference group); 95% CI, 95% confidence interval (A 95% confidence interval means that 95% of the values of an unobservable parameter of interest would set in the interval if the experiment is repeated); na, not available; ref, reference

* Significant p-value

^a^ pathological tumor (pT) category refers to the stage (i.e. size and extent) of primary tumor in the TNM tumor staging system. pT2 = Tumor confined within prostate, pT3 = Tumor invading extraprostatic tissue and/or seminal vesicle(s).

^b^ N category describes in TNM system weather (N1) or not (N0) the cancer has spread to the regional lymph nodes.

**Fig 1:**
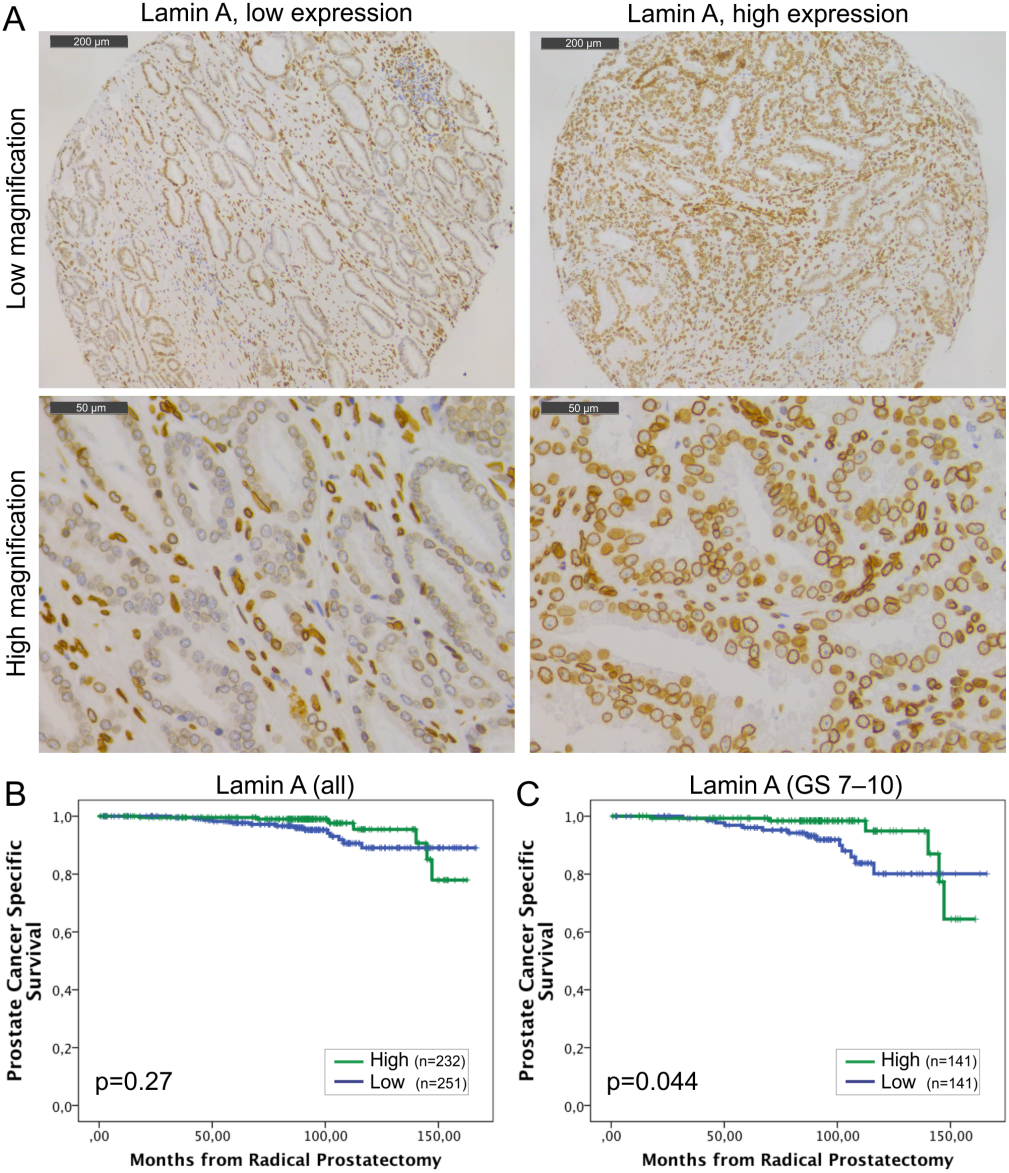
Low lamin A expression in PCa predicts unfavorable DSS. (A) Representative examples of TMA slides stained for lamin A with immunohistochemistry. Low and high power field images from both low and high expressing tumors are shown. (B-C) Kaplan-Meier analysis shows a trend between low lamin A expression and poor DSS in the entire cohort (B; p = 0.27). However, in the subpopulation of patients with Gleason score >6 tumors, there is statistically significant difference (C; p = 0.044).

Staining with lamin A specific antibody showed that lamin A is expressed in both basal and luminal epithelial cells of normal prostatic glands and is enriched at the nuclear lamina region as expected (Fig 1A). However, there were significant differences in staining intensities between carcinomas from individual patients (from grade 0 to 3). The mean staining intensities of carcinoma cell nuclei were significantly higher when compared to normal epithelium in benign samples from the same patients (paired-samples t-test; mean staining intensities +/- SEM 1.97+/-0.03 and 1.45+/-0.03 for carcinoma and benign, respectively, p<0.001). Low lamin A expression (grade 0–1) was statistically significantly associated with lymph node positivity (Pearson Chi-Square test, p = 0.009) but no other clinicopathological variables tested. In Kaplan-Meier estimation analysis, there was no correlation between lamin A expression and BCR (data not shown). When DSS was used as an end point, a trend between low lamin A expression and poor outcome was seen in the whole population but this difference failed to show statistical significance (Fig 1B; p = 0.27). However, in the subpopulation of GS >6 tumors with potentially aggressive behavior, low lamin A expression was statistically significantly associated with poor outcome (Fig 1C; p = 0.044). These results suggest that diminished amount of lamin A may promote lymphovascular invasion and progression of potentially metastatic disease.

Lamin C is another major A-type lamin encoded by *LMNA* through alternative splicing. This isoform is 92 amino acids shorter than lamin A and carries a unique C-terminal tail that is removed from pre-lamin A during posttranslational modification. The staining with lamin C antibody (detecting the lamin C specific C-terminal tail domain only) showed that lamin C is similarly expressed and present at the nuclear lamina in both normal basal and luminal epithelial cells as well as in the majority of (but not all) carcinomas with low to moderate intensities (S1A Fig). A paired T-test showed that the mean staining intensities for lamin C were significantly higher in carcinoma samples (1.30+/-0.06) relative to normal epithelium (1.04+/-0.05, p = 0.001). There was no correlation between lamin C expression and clinicopathological variables (data not shown). In the Kaplan-Meier analysis, we found no correlation between lamin C expression and BCR (data not shown) similar to lamin A. There was a trend between undetectably low lamin C expression (grade 0) and less favorable DSS when patients with all the different Gleason groups were studied (S1B Fig; p = 0.14). Moreover, in the GS >6 subpopulation the patients with lamin C negative tumors (grade 0) had statistically significantly higher risk to die from PCa (S1C Fig; p = 0.009). These results suggest that lamin A and C, both produced from *LMNA* gene, may have similar biological effects in malignant transformation of PCa.

**Fig 2:**
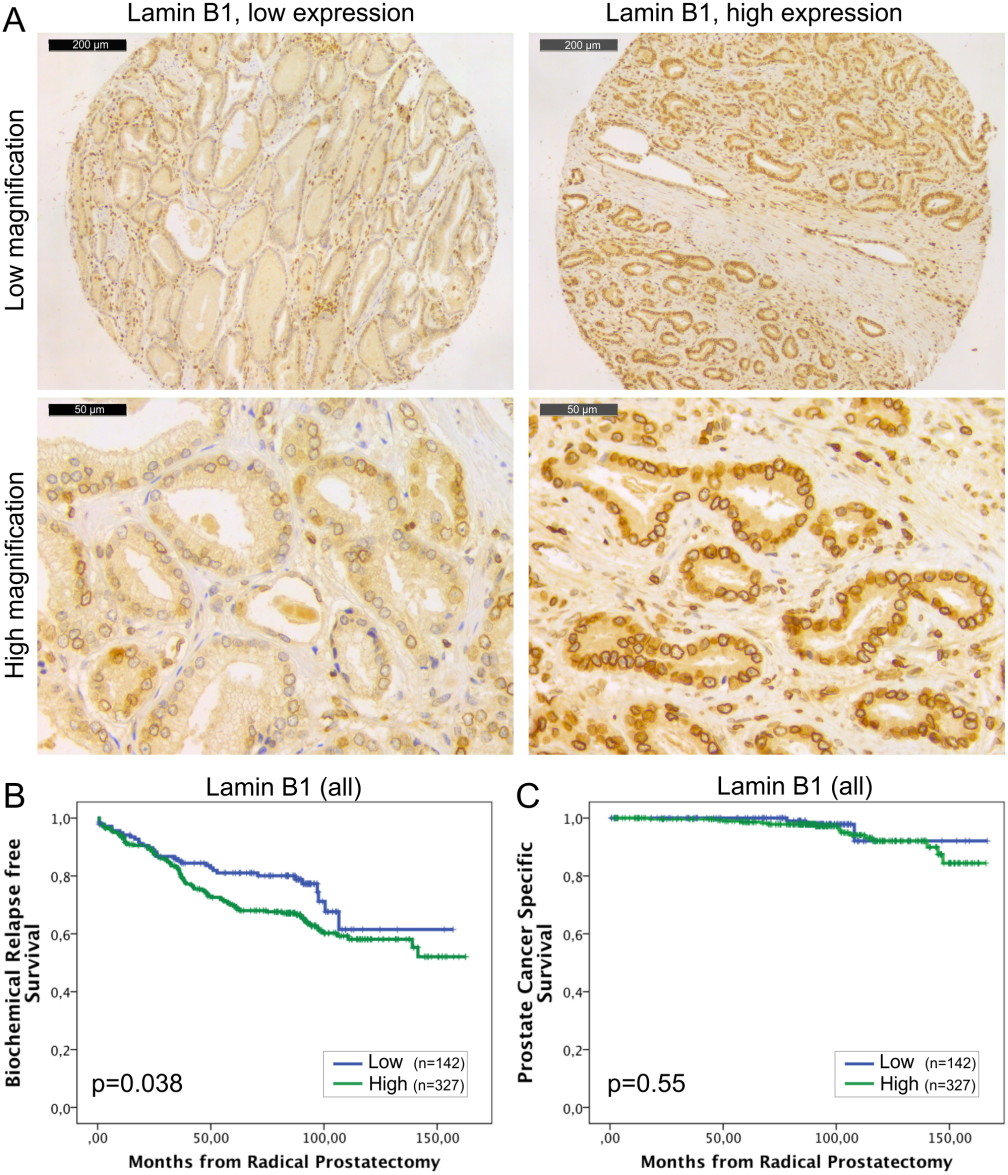
High lamin B1 expression in PCa predicts increased risk for BCR. (A) Representative examples of TMA slides stained for lamin B1 with immunohistochemistry. Low and high power field images from both low and high expressing tumors are shown. (B-C) Kaplan-Meier analysis indicates that high lamin B1 expression predicts shorter time to BCR in the whole cohort (B; p = 0.038) but has no correlation with DSS (C; p = 0.55).

The expression of lamin B1 is generally detected in all the eukaryotic cells and its loss has severe consequences during embryogenesis as highlighted with developmental abnormalities of lung and brain and early perinatal death in *LMNB1* knockout mice [22]. In our material, all the histologically benign samples showed mild to moderate staining intensity for lamin B1. All the carcinoma samples were lamin B1 positive as well (Fig 2A) but 65% of the carcinomas showed high expression (grade 3) of lamin B1. Paired t-test revealed that there was significantly more lamin B1 staining in carcinomas relative to normal benign samples (2.7+/-0.03 vs. 1.7+/-0.03, p<0.001). No significant correlation was found between lamin B1 expression and clinicopathological variables tested. In the Kaplan-Meier analysis, high expression of lamin B1 was associated with BCR in the whole cohort (Fig 2B; p = 0.038) and in the subpopulation of GS >6 (p = 0.034, data not shown). However, there was no correlation between lamin B1 expression and DSS in either the whole cohort (Fig 2C; p = 0.55) or in the subpopulation of patients with GS >6 tumors (p = 0.57, data not shown). In multivariable Cox regression analysis high lamin B1 expression remained an independent predictor of BCR in the entire cohort when adjusted for preoperative PSA and Gleason grade (HR 1.8, 95% CI 1.1–2.9, p = 0.023; Table 3). These results suggest that high expression of lamin B1, for unknown reasons, increases risk for PCa recurrence but has no significant effect on PCa survival.

**Fig 3:**
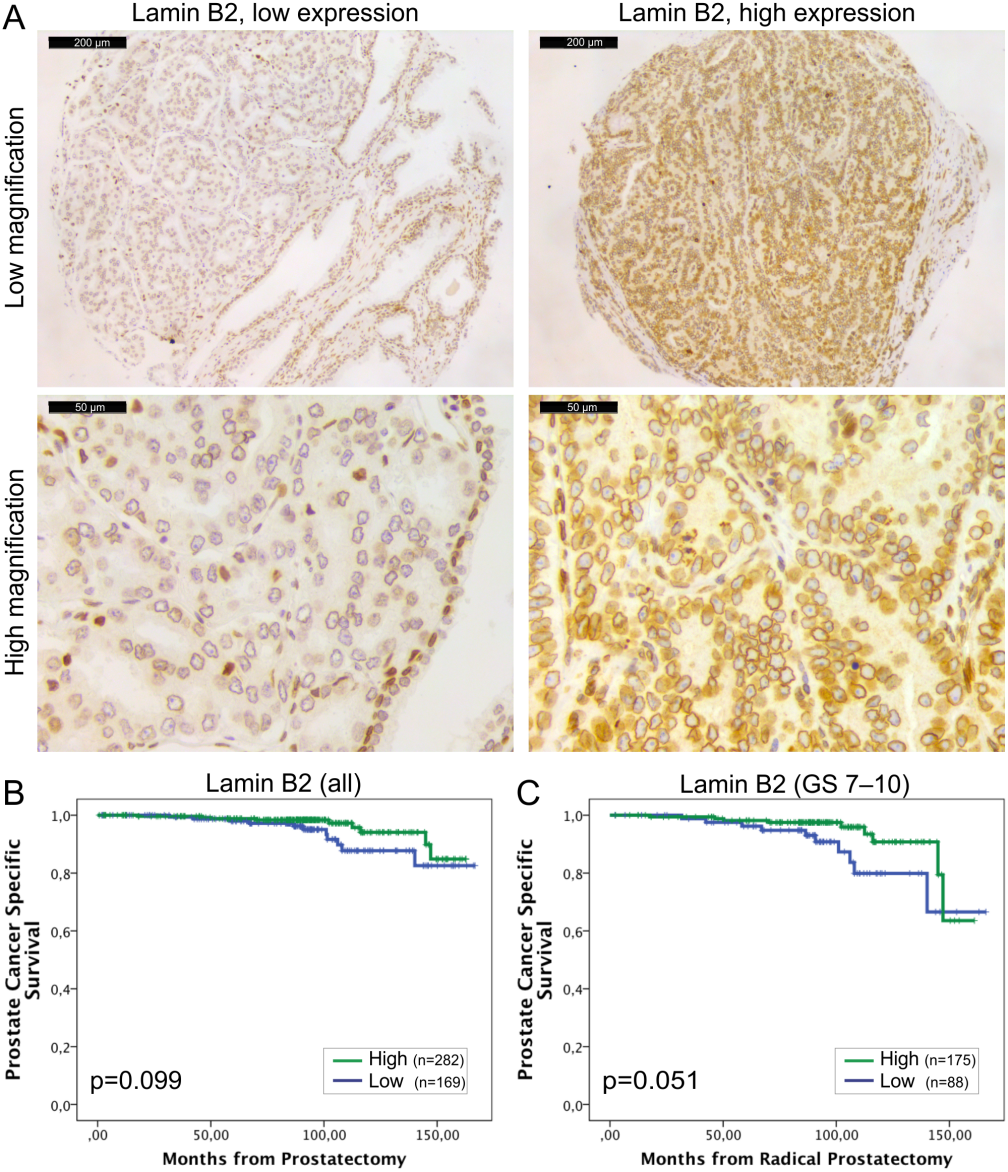
Low lamin B2 expression in PCa predicts shortened DSS. (A) Representative examples of TMA slides stained for lamin B2 with immunohistochemistry. Low and high power field images from both low and high expressing tumors are shown. (B-C) Kaplan-Meier analysis shows a trend between low lamin B2 expression and shortened DSS in the entire cohort (B; p = 0.099). In the subpopulation of patients with Gleason score >6 tumors, Kaplan-Meyer analysis slightly fails to show statistically significant difference but a strong trend is seen (C; p = 0.051).

Lamin B2 was expressed in all the normal and malignant prostate glands (Fig 3A). However, similar to other lamins, there was statistically significantly higher IHC intensity of lamin B2 in the cancerous glands relative to normal benign glands (1.74+/-0.03 vs. 1.48+/-0.03, p<0.001). Low lamin B2 expression was associated with lymph node positivity (Chi-square; p = 0.005) and there was a trend between low lamin B2 expression and T category (p = 0.076) and EPE (p = 0.098). In Kaplan-Meier analysis, there was no correlation between lamin B2 expression and BCR (data not shown). However, When DSS was used as an end point, there was a trend between low lamin B2 expression and poor DSS in the whole cohort (Fig 3B; p = 0.099) and a borderline statistical significance in GS >6 subpopulation (Fig 3C; p = 0.051). These results show that lamin B1 and lamin B2 predict different outcome in PCa which may be due to differential roles in cancerous prostate cells.

## Discussion

The nuclear lamins are highly conserved among species and phylogenetic analysis suggests that they represent ancestors of whole IF protein family [23]. There is accumulating evidence that lamins not only support physical shape of the nucleus but also contribute several fundamental nuclear functions such as genome organization and transcription through interactions with chromatin and multiple transcription factors [7]. Dysfunction in these mechanisms may have deleterious effects *in vivo* as evidenced by various hereditary human diseases due to lamin mutations [9]. However, the role and alterations of lamins in malignant diseases are less well understood.

In the present study we investigated the expression of different lamins in PCa and their potential use in estimation of patient outcome using an extensive TMA material and a detailed clinical patient data with a median follow-up time of more than 8 years. While the expression of all the major lamins was detected in prostatic epithelium, the statistical analysis revealed some interesting changes that are relevant in the light of recent *in vitro* findings about lamin function. Swift et al. have shown that the amount of lamin A and the ratio of A- and B-type lamins correlates with tissue stiffness (e.g. muscle has more lamin A than brain) to stabilize nucleus and chromatin from physical stress [24]. Furthermore, low levels of A-type lamins increase nuclear deformability and enhance cell migration through microfluidic constrictions *in vitro* [25] and *in vivo* in a mouse tumor model [26]. This is consistent with our results showing that reduced amounts of lamin A/C are associated with lymph node metastasis and unfavorable DSS, presumably due to increased lymphovascular invasion. The association between low lamin A expression and unfavourable DSS was statistically significant in Kaplan-Meier analysis carried out on patients with potentially aggressive GS>6 disease (Fig 1C). However, in the multivariate Cox regression analysis, that also takes other clinicopathological variables into account, lamin A slightly failed to reach statistical significance (p = 0.052, Table 4) suggesting that it is not an independent predictive marker different from lamin B1. The data from others suggest that lamin A is overexpressed in the invasive front in PCa and may increase local migration and invasion [16]. If this were the case, one would expect that high expression levels of lamin A/C correlate with BCR but we were unable to confirm such phenomenon. These differences may be, at least partly, due to variation in sampling. Despite the fact that we obtained three triplicate samples from the index tumor, these tissue cores represent overall tumor expression levels and were not intentionally targeted to areas of invasive front.

The results from this and other studies suggest that the changes in lamin expression and their impact on tumor biology and clinical outcome may be complex and organ-specific. Previously the prognostic value of lamins in cancer progression has been established in colorectal cancer (CRC) where lamin A expression predicts poor outcome when compared to lamin A negative cancers [12]. However, little is known about the prognostic role of lamins in other solid tumors and our results suggest opposite role for lamin A in PCa progression (Fig 1C). A fundamental difference between these two types of adenocarcinomas is that 30% of CRCs were virtually devoid of lamin A [12] but at least some lamin A expression was detected in all PCas included in the present study. The results from *in vitro* studies suggest that silencing of lamin A may actually sensitize the cells for mechanical stress and reduce cell survival [26]. Therefore, presence of lamin A in CRC may mechanically protect the migrating cancer cells and reduce tumor cell death when compared to lamin A negative tumors while in the case of PCa moderately low amount of lamin A may simultaneously provide an ideal nuclear deformability and sufficient protection against mechanical stress in invading cells.

A- and B-type lamins have differential roles in normal cells. While A-type lamins are mostly expressed in terminally differentiated cells, one or both of the B-type lamins are expressed in all animal cells. Mice lacking lamin B1, lamin B2 or both have defects in lung and brain development and these mice die at birth suggesting an essential developmental function [22]. Interestingly, increased production of one B-type lamin does not rescue the loss of the other B-type lamin indicating that lamin B1 and B2 have also differential cellular functions [27]. Similarly, our results suggest differential roles for lamin B1 and B2 in PCa progression. PCa tissues contained significantly more lamin B1 than benign tissues from the same patients. High lamin B1 expression associated with BCR in the entire cohort, as well as in the GS>6 subpopulation (Fig 2B), and in multivariable Cox regression analysis lamin B1 appeared an independent prognostic factor for BCR (Table 3). Nevertheless, there was no statistically significant correlation between lamin B1 expression and DSS (Fig 2C and Table 4). Although our cohort is relatively large, it is important to notice that only 4.4% of patients died from PCa during the follow-up while 31% showed BCR. In most cases, the latter may be due to minimal local residual tumor that has no impact on DSS after salvage radiation therapy [28]. Therefore, high lamin B1 expression is more likely promoting local growth and/or proliferation, rather than enabling metastatic behavior through lymphovascular invasion. In support for this, a recent study has shown that moderate reduction of lamin B1 in cancer cells delays cell cycle progression [29]. Furthermore, silencing of lamin B1 in normal diploid WI-38 lung cells causes replicative senescence while overexpression increases the proliferation rate [30]. Coradeghini et al. have previously reported that lamin B1 expression correlates with GS and could be used as a biomarker in tumor differentiation and prognostics [17]. We were unable to confirm the correlation with GS but even more importantly our results clearly suggest that lamin B1 is an independent predictor that could be used to determine the risk of local growth and proliferation.

Little is known about role of lamin B2 and its association with cancer. In the present study, low lamin B2 expression predicted LN positivity, similar to lamin A and C. Additionally, there was a trend between low expression of lamin B2 and less favorable DSS (Fig 3B and 3C) but little in common with lamin B1. Recently, Kuga et al. provided evidence that lamin B2 plays a role in mitotic spindle formation [31]. Furthermore, knockdown of lamin B2 resulted in chromosomal instability in CRC cells [31]. Although a more precise role of lamin B2 in spindle function still needs to be addressed it is tempting to speculate that PCa tumors with low amounts of lamin B2 are more prone to chromosomal instability, aneuploidy and eventually progression towards aggressive and potentially metastatic phenotype.

In summary, our results suggest that lamins have differential roles in PCa progression and that they could be used as prognostic biomarkers in PCa diagnostics and risk stratification. For example, immunohistochemical lamin A staining from prostatic biopsy material might help in identifying the high risk patient who would benefit from lymph node dissection. However, the use semi-quantitative methods like immunohistochemistry in routine clinical diagnostic work may be challenging and would require sensitive and well validated control samples. Due to relatively high frequency of BCR and low frequency of mortality in PCa, further studies with even more extensive and multi-centered patient cohorts would be needed to validate the prognostic role of lamins in PCa. In addition, it would be of great interest to study the expression of A- and B-type lamins in PCa LN and distant metastases in the following studies.

## Supporting Information

S1 Fig Low lamin C expression in PCa predicts shortened DSS. (A) Representative examples of TMA slides stained for lamin C with immunohistochemistry. Low and high power field images from both low and high expressing tumors are shown. (B-C) Kaplan-Meier analysis shows a trend between low lamin C expression and shortened DSS in the entire cohort (B; p = 0.14). However, in the subpopulation of patients with Gleason score >6 tumors, there is statistically significant difference (C; p = 0.009).
(TIF)
