# Supplementary figures and images for: Differential Predictive Roles of A- and B-Type Nuclear Lamins in Prostate Cancer Progression
Source: PLoS One. 2015 Oct 15;10(10):e0140671. doi: 10.1371/journal.pone.0140671 (PMC4607298; doi:10.1371/journal.pone.0140671)

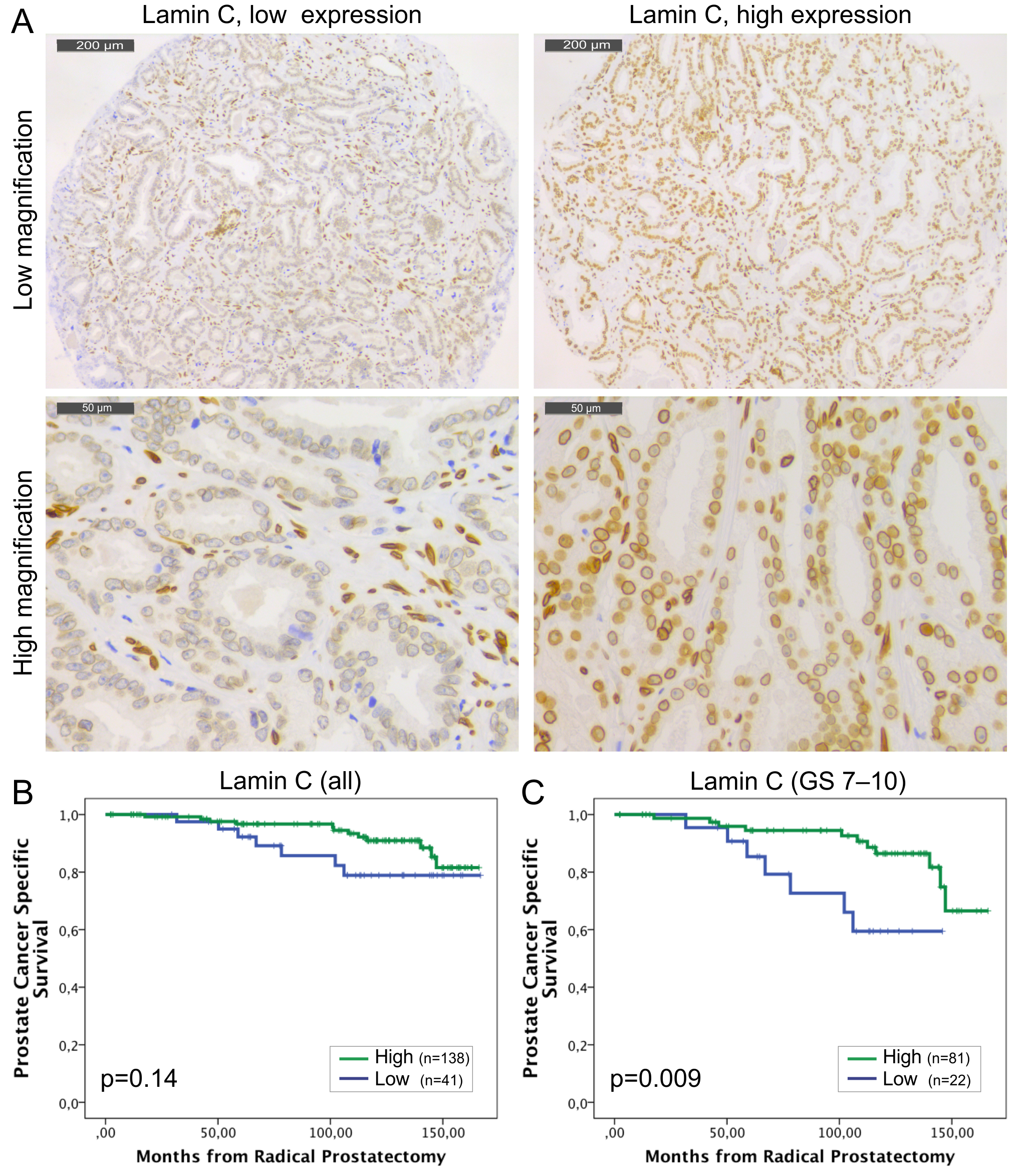

Supplement: S1 Fig — (A) Representative examples of TMA slides stained for lamin C with immunohistochemistry. Low and high power field images from both low and high expressing tumors are shown. (B-C) Kaplan-Meier analysis shows a trend between low lamin C expression and shortened DSS in the entire cohort (B; p = 0.14). However, in the subpopulation of patients with Gleason score >6 tumors, there is statistically significant difference (C; p = 0.009). (TIF) [file pone.0140671.s001.tif]
